# New World dung beetle (Coleoptera: Scarabaeidae: Scarabaeinae) colonization of a recent Miocene insular territory: The case of Costa Rica

**DOI:** 10.1002/ece3.11436

**Published:** 2024-05-30

**Authors:** Bert Kohlmann, Renato Portela Salomão, Ángel Solís

**Affiliations:** ^1^ BioAlfa Barcoding Project Santo Domingo de Heredia Costa Rica; ^2^ Facultad de Estudios Superiores Iztacala Universidad Nacional Autónoma de México Tlalnepantla de Baz Mexico; ^3^ Programa de Pós‐graduação Em Ecologia Instituto Nacional de Pesquisas da Amazônia Manaus Brazil

**Keywords:** ecological equivalents, Elevational Rapoport Rule, insular syndrome, Miocene colonization, montane diversification, temporal overlap area

## Abstract

Costa Rica emerged from the seas as a new geological territory during the Miocene as an insular archipelago. It later became part of a continental area once it became a segment of Central America. Two dung beetle genera that colonized this new territory from South and North America, *Canthidium* and *Onthophagus* (Coleoptera: Scarabaeidae: Scarabaeinae), are here studied, in the first analysis of a volcanic paleo‐archipelago, colonized from its emergence, and then later becoming *terra firma*. To assess their biodiversity distribution patterns, we analyzed the effect of biogeography, ecosystem origins, and body size on their altitudinal distribution patterns in three geographic basins of Costa Rica. Based on 32 years of collecting representing more than 158,000 specimens from 1017 localities, we undertook Generalized Linear Models of the two dung beetle genera to assess the effects of biodiversity and biogeographical distribution patterns. *Canthidium* and *Onthophagus* species ranged from 0 to 3000 m a.s.l., with an abrupt diversity decline at altitudes above 1500 m. Endemic species tended to show a higher altitudinal mean with a narrow altitudinal band distribution than non‐endemic dung beetle species. Although there was a trend of decreasing species body size with the increase in altitude, such a trend depended on the distribution pattern of the species group. This possible insular‐mediated endemicity mechanism has generated baffling biodiversity levels, considered the highest worldwide per unit area. Costa Rica is an expanse represented by a geographic overlap of two or more temporally disjunct areas and is not part of a natural transition zone. The effect of the insular Miocene origin of Costa Rica still pervades today, reflected by different insular syndromes shown by the dung beetle fauna. The importance of geological origins in generating biodiversity seems to have been an underrated criterion for conservation biology practices and should be considered ex officio.

## Graphical abstract

In Costa Rica, Canthidium and Onthophagus have abrupt diversity declines above 1500 m a.s.l. Endemic species showed narrower vertical distribution when compared to non‐endemic ones.

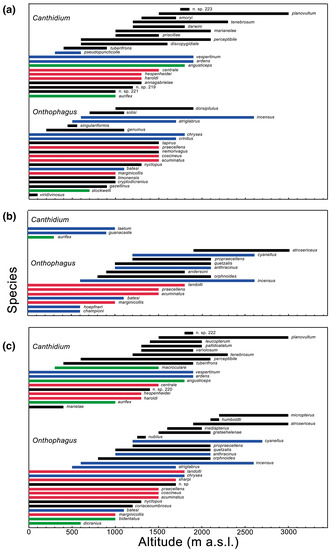

Es gibt ein Ziel, aber keinen Weg; was wir Weg nennen, ist Zögern.

—Kafka, Die Zürauer Aphorismen (1917–18)(There is a destination but no path; what we call path is hesitation.)

## 1 INTRODUCTION

Species distribution patterns represent an essential knowledge determinant for many biological, theoretical, and practical areas, such as macroecology, biogeography, and conservation biology (Isaac et al., 2020). Large‐scale studies encompassing an entire country may allow broader comprehension of species distribution patterns compared to more regional studies. Nonetheless, interpreting spatial trends is often context‐dependent under broad scales due to the wide variety of ecosystem and landscape contexts (i.e., complex systems; see Newman et al., 2019). The species' geographical origin comprises important species traits to obtain precise distribution patterns, giving detailed cues regarding the species' historical spatial process at broad scales (Buckley & Catford, 2016; Trethowan et al., 2022). Moreover, important insights have been depicted when considering body size in spatial distribution dynamics, as the effect of latitude on species body size, as well as filtering small‐bodied species in higher altitudes compared to lowlands (Kohlmann et al., 2021; Meiri & Dayan, 2004; Tseng & Pari, 2019). In this sense, species body size and geographical origin may synergically affect species distribution patterns (Blackburn & Gaston, 1996; Kohlmann et al., 2021).

Among the determinant drivers of species distribution, elevation is a widely studied theme, and diverse theories have been proposed to explain how species distribute throughout mountain altitudinal gradients (e.g., Alvarado et al., 2020; Kohlmann et al., 2021; Lobo & Halffter, 2000; Sun et al., 2020). The relationship between species' body size and altitude is context‐dependent (Salomão et al., 2021; Sun et al., 2020). Due to limiting environmental conditions found in highlands compared to lowlands, species diversity tends to decrease in higher altitudes compared to lower altitudes (Alvarado et al., 2020; Perillo et al., 2017; but see Pôrto et al., 2004). If horizontal colonization dominates, the gradient of reduction in species richness is attenuated, whereas if vertical colonization dominates then the reduction in richness is greater (Joaqui et al., 2021; Lobo & Halffter, 2000). In America, numerous mountain chains are determinants for biogeographic domains, like the Andean Mountain chain, which limits species dispersal and affects species geographical distribution patterns (Lizardo et al., 2022, 2024; Morrone, 2006, 2020). Among them, the mountain chains located in Southern North America and Central America (such as the Mexican Transverse Neovolcanic Belt) have a noteworthy role in limiting the distribution of many taxa and thus separating Neotropical and Nearctic domains (Lizardo et al., 2022, 2024; Morrone, 2006, 2020). As mountains are determinant for this biogeographic separation, it is crucial to assess the role of altitude in the distribution of the species in such regions, which can give cues regarding spatial processes that drive biodiversity patterns in the American continent.

Costa Rica belongs to the Middle American biodiversity hotspot, one of the 36 world hotspots defined by Mittermeier et al. (2004). Costa Rica has 51,180 km^2^ of continental and insular land surface, representing 0.03% of the Earth's surface (Jiménez, 1995; MAE, 2000). In the ranking of world diversity, Costa Rica occupies the 20th place, approximately (Ávalos, 2019). From a biodiversity perspective, what makes Costa Rica special is its species density (number of species per unit of area; Kappelle, 2016). Using this measure, Costa Rica could probably occupy the first place in the world regarding biodiversity (Kappelle, 2016). This country possesses approximately 5% of the described world diversity (about 191,235 described species so far; Ávalos, 2019). To give a comparative idea of species density, Costa Rica registers 209.3 plant species per 1000 km^2^, whereas Colombia, in the second world place, records only 43.8 plant species per 1000 km^2^ (Ávalos, 2019).

Dung beetles (Coleoptera: Scarabaeinae) have been particularly well studied for their systematics, distribution, and ecology in Costa Rica during the last 32 years (e.g., Kohlmann et al., 2010; Kohlmann & Wilkinson, 2007; Solís & Kohlmann, 2012). Costa Rica is the best‐studied and collected American country concerning dung beetles (Cupello et al., 2023). Dung beetles are considered to represent a good indicator group (Halffter & Favila, 1993; Nichols et al., 2007; Spector & Forsyth, 1998) and an excellent model to assess biodiversity distribution patterns (Kohlmann et al., 2021; Salomão et al., 2021). Among dung beetle groups, the genera *Canthidium* Erichson, 1847 and *Onthophagus* Latreille, 1802 comprise two of the most studied ones in this region and the two most species‐rich dung beetle genera in Costa Rica (Cupello, 2018; Cupello et al., 2023; Kohlmann & Solís, 2001; Solís & Kohlmann, 2004, 2012). Due to the large amount of information regarding these genera, they can serve as excellent proxies to clarify geographical distribution trends of tropical and subtropical biodiversity in the Americas.

This study aims to assess the biodiversity distribution patterns of *Canthidium* and *Onthophagus* in Costa Rican landscapes, analyzing the effect of biogeographical and geological origins and body size on altitudinal distribution patterns (species altitudinal range and mean; see Kohlmann et al., 2021) of dung beetles in three geographic basins of Costa Rica (Caribbean, North Pacific, and South Pacific). *Onthophagus* has a possible Afrotropical origin having migrated through the Behring Strait into America, while *Canthidium* has a Neotropical (non‐Gondwanan) origin (Breeschoten et al., 2016; Cupello, 2018). Harold had already indicated that *Canthidium* represents the Onthophages in Tropical America (“…*Canthidium*…, welche im tropischen Amerika als die Repräsentanten der Onthophagen erscheinen, …”; [Harold, 1867: p. 1]), suggesting they both are ecological equivalents (Solís et al., 2024). Both genera must have colonized the initial Costa Rican Miocene Island archipelago that emerged from the sea, an archipelago that was settled by North and South American biota at different times (Alvarado & Cárdenes, 2016; Coates, 1997; Cox et al., 2020; Lomolino et al., 2017; Solís et al., 2024). The process we try to elucidate in this study is whether two genera of different biogeographical origins but akin ecologically generate similar/dissimilar altitudinal species distribution patterns while having colonized a new geological territory of Miocene insular origin.

## 2 MATERIALS AND METHODS

### 2.1 Study site

**FIGURE 1:**
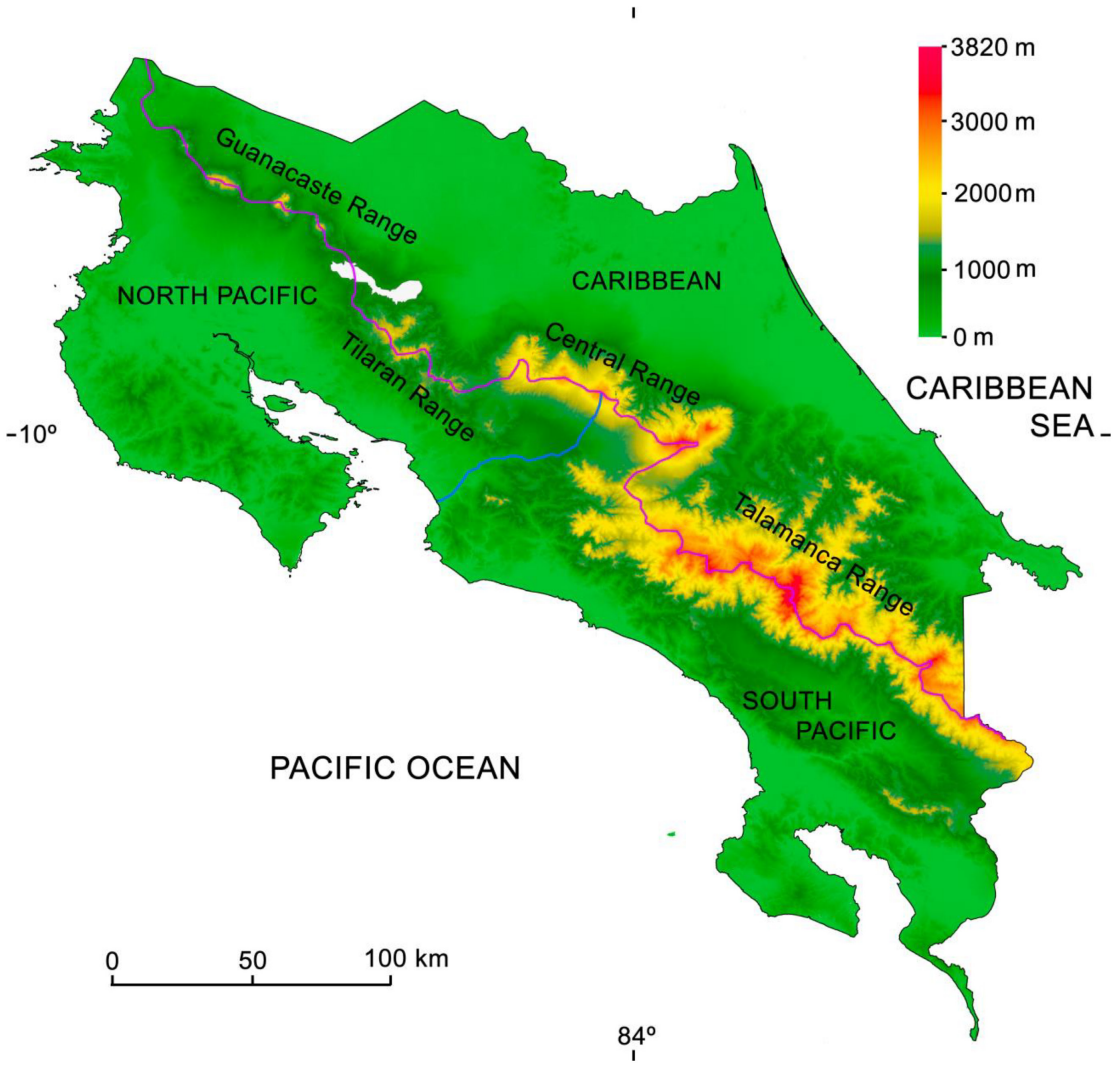
General map of Costa Rica showing the main mountain ranges and the three geographic regions. The red line represents the *divortium aquarum*, and the blue line represents the Grande de Tárcoles River, separating the North from the South Pacific basins.

Costa Rica is neatly divided into two watersheds by a central mountain chain formed by four mountain ranges, encompassing three geographic basins, a Caribbean, and a North Pacific and a South Pacific one (Figure 1). Starting from the NW to SE, we have the active volcanic Guanacaste Range (approximately 80 km length, 1100–2028 m a.s.l.; volcanoes of Quaternary origin), the extinct volcanic Tilarán Range (approximately 40 km length, 881–1020 m a.s.l.; Miocene‐Lower Pleistocene origin); the active volcanic Central Range (approximately 86 km length, 2024–3423 m a.s.l.; volcanoes of Quaternary origin); finally, the Talamanca Range (approximately 320 km length, 3084–3812 m a.s.l.; Miocene origin), showing glacial and periglacial Pleistocene geomorphology (Alvarado & Cárdenes, 2016). Regarding soil use, the Caribbean is a vital banana and pineapple producer; the North Pacific produces rice, sugarcane, and watermelons; the South Pacific is a rice and African palm oil producer (Kohlmann et al., 2002). The cloud forest Pacific area (which is located between 1200 and 1900 m a.s.l.) is devoted to the production of coffee, and the volcanoes of the Cordillera Central specialize in the production of dairy products, ornamental ferns, strawberries, and potatoes (Kohlmann et al., 2002).

The Caribbean basin (3 to 25°C mean annual temperature; from 2000 to 8000 mm annual rain slope; 24,258 km^2^; Figure 1) is always wet due to the westerly air masses moving from the Caribbean Sea. The driest months go from February to March, sometimes the first weeks of April, and it rains from April to December with a short dry spell around September and October. The lowlands (ranging from 0 to 500 m a.s.l.) contain lowland moist and wet forests and large areas of swamp forest. The foothills and uplands (from 500 to 1500 m a.s.l.) have tropical wet and rainforests. The highlands (1500 to 3840 m a.s.l.) present montane moist and wet forests, rainforest, and subalpine pluvial paramo (alpine meadow). The Caribbean and South Pacific represent basins where the present tropical rainforest is considered a recent invasion from South America into the Central and North American areas, dating from around 10,000 years ago (Colinvaux, 1997; Gómez, 1986; Toledo, 1982). However, a tropical rainforest has occupied the Costa Rican area since the Miocene (~23 to ~5 Ma), probably having had a different community structure from the present tropical rainforest (Burnham & Graham, 1999; Colinvaux, 1997).

The North Pacific (8 to 27°C mat; ranging from 1100 to 4000 mm annual rain slope; 13,632 km^2^; Figure 1) basin has a well‐defined seasonality with a rainy season extending from May to October and a prolonged dry season from November to May. General biological activity is correlated with rainfall seasonality in this area. The lowlands (0 to 600 m a.s.l.) are covered by dry tropical forest. The foothills and uplands (600 to 1600 m a.s.l.) present a moist, premontane wet forest and rainforest. The highlands (ranging from 1600 to 3343 m a.s.l.) present lower montane and montane moist and wet forests and rainforests and a small area of subalpine pluvial *páramo*. The dry tropical forest of the North Pacific seems to have expanded its distribution into Costa Rica around 2.5 Ma coming from Mexico (Becerra, 2005; Graham & Dilcher, 1995; Pérez‐García et al., 2012).

The South Pacific basin (3 to 27°C mat; from 2000 to 8000 mm ap; 13,145 km^2^; Figure 1) experiences a short dry season, but never as in the North Pacific. The lowlands (from 0 to 600 m a.s.l.) present lowland moist and wet forests. The foothills and uplands (600 to 1600 m a.s.l.) are covered by premontane wet and rainforests. The highlands (1600 to 3840 m a.s.l.) have montane, moist, wet‐and rainforests, and subalpine pluvial páramo. The rainforest from the South Pacific basin became partially isolated from the Caribbean and South American rainforest during the Late Miocene thanks to the uplift of the Talamanca Range, which had already formed a well‐defined mountain range by 8–6 Mya (Alfaro et al., 2021; Alvarado & Gans, 2012).

### 2.2 Taxa

Information regarding dung beetle distribution was taken from an electronic database accumulated by A. Solís using as information sources material from the ex‐INBio entomological collection (now in the Museo Nacional de Costa Rica); from the Naturkundemuseum Berlin; Senckenberg Naturhistorische‐Sammlungen, Dresden; Zoologische Staatssammlung München, Munich; Musée National d'Histoire Naturelle, Paris; British Museum of Natural History, London; American Museum of Natural History, New York; Smithsonian Institution, Washington and Panama; Canadian Museum of Nature and Canadian National Collection, Ottawa; Universidad de León and Jean Michel Maes Collection, León, Nicaragua; Ángel Solís Collection and Bert Kohlmann Collection, Costa Rica. This information accumulated over a period of 32 years, representing more than 158,000 specimens from 1017 localities throughout the country. The data that allows replicability of the research is provided in the Appendix S1 of this study.

To have the best possible representation of species numbers of the genera under study, we have included five new cryptic *Canthidium* and one new *Onthophagus* species detected as part of an ongoing project doing a mtDNA analysis of all Costa Rican dung beetle species (Solís & Kohlmann, 2012). Their formal description is to follow in another paper. We recorded 488 collecting localities for *Canthidium* (Caribbean: 202, North Pacific: 66, South Pacific: 220) and 610 for *Onthophagus* (Caribbean: 229, North Pacific: 92, South Pacific: 289). Dung beetle records were sorted throughout the altitudinal bands of each geographic study region (Caribbean, North Pacific, South Pacific), in which a 100 m‐interval was used to stratify the vertical distribution of dung beetle species. Therefore, we had a total data of 90 different altitudinal bands (30 for the 3000 m a.s.l. altitude of the North Pacific region, 30 for the 3000 m a.s.l. altitude of the South Pacific, 30 for the 3000 m a.s.l. altitude of the Caribbean region).

The genus *Onthophagus* is a hyperdiverse taxon. Schoolmeesters (2023) has so far listed 2255 described species. The New World *Onthophagus* fauna includes about 226 species, 41 from Costa Rica (Cupello et al., 2023; Moctezuma & Halffter, 2021; Solís & Kohlmann, 2012). American *Onthophagus* is generally considered to belong to only one worldwide subgenus (Zunino & Halffter, 1981, 1988, 1997, 2019), *Onthophagus* sensu stricto, as defined by Zunino (1979). Emlen et al. (2005), Tarasov and Solodovnikov (2011), Breeschoten et al. (2016), and Schwery and O'Meara (2021) have proposed that the American Onthophagini are a monophyletic group. On the contrary, Zunino (1979), Palestrini (1980), Varola (1980), Zunino and Halffter (1981, 1997), Palestrini (1985), Palestrini and Zunino (1986), and Howden and Gill (1993) have considered that American Onthophagini are derived from several invasions stemming from the Oriental and Palearctic Regions, especially from the Chinese Transition Zone.

The genus *Canthidium* contains 178 species listed in two subgenera, *Canthidium* and *Neocanthidium* (Cupello et al., 2023). Costa Rica registers 30 species at present. It is a genus of Neotropical (South American) origin that has colonized the southern part of North America, penetrating in a limited way into Arizona and Texas (Kohlmann & Solís, 2006). Cupello (2018) indicates that this genus has the most elusive systematics. The ecological equivalency mentioned above could probably explain why *Onthophagus* has not speciated as intensively in South America as in North and Central America and vice versa *for Onthophagus*.

Regarding phylogenetic analyses, none have been done for *Canthidium*, but there are a few for *Onthophagus*. Three of them are important (Breeschoten et al., 2016; Emlen et al., 2005; Schwery & O'Meara, 2021) because they suggest that the *Onthophagus clypeatus* species group, which is exceptionally biodiverse in Costa Rica, was the first American *Onthophagus* group to appear in the American Continent. The study by Breeschoten et al. (2016) proposes that the *Onthophagus clypeatus* species‐group branched around 20.5 Ma, coinciding with the emergence of Costa Rica as an island arch during the Miocene. Moctezuma et al. (2024) propose a much younger age of around 12 Mya for this same group. Breeschoten et al. (2016) suggest a subsequent branching of other *Onthophagus* species groups: landolti (~17 Ma) and hircus (~14 Ma); both groups are represented in Costa Rica by taxa that have a widespread distribution in North and South America.

### 2.3 Geology

Geological evidence has suggested that at 25–13 Ma, a magmatic island arc extended from Panama to the present Caribbean side of Nicaragua, called the Talamanca‐Sarapiquí volcanic arc in Costa Rica (Alvarado & Cárdenes, 2016; Coates, 1997; Solís et al., 2024). The configuration of the emerged territory of Costa Rica and Panama began to take a form that gradually approached its current geography. The closure of the Nicaragua portal establishing a land connection of insular Costa Rica with Nicaragua, and thus to Nuclear Central America, seems to have taken place around 5.8–5.0 Ma (Solís et al., 2024). This event can be considered the early beginning of the Central American Isthmus's closure, even though the definitive closure of the Panama portal occurred around 2.8 Ma (Montes et al., 2015; O'Dea et al., 2016). This arc was almost continuously active, with volcanic activity of variable intensity until the present. Its formation likely began through the origin of underwater volcanoes, which gradually transformed into volcanic islands active and inactive; they eventually fused into a volcanic archipelago through tectonics (Alvarado & Cárdenes, 2016; Solís et al., 2024).

The Costa Rican entomofauna shows typical island syndromes (Baeckens & van Damme, 2020; Matthews & Triantis, 2021), like Foster's rule (Foster, 1964; van Valen, 1973), where the endemic *Canthidium* species are smaller than the non‐endemic ones, and the endemic *Onthophagus* species are larger than the non‐endemic ones (*p* = .035; *p* = .028, respectively; Solís et al., 2024). This phenomenon was first noted by Howden and Young (1981) for the endemic *dung beetle* species of Eastern Costa Rica and Panama (*p* = .07).

Other island syndromes in Costa Rica are the generation of brachyptery in insects, also known as “Darwin's Factor” (Darwin, 1859; Lawrence et al., 1991) and their concomitant microendemic distribution (Gillespie & Roderick, 2002). In Costa Rica, one species of *Canthidium* (*C. planovultum* Howden & Young, 1981) and three of *Onthophagus* (*O. humboldti* Kohlmann, Solís and Alvarado, 2019; *O. inediapterus* Kohlmann & Solís, 2001; *O. micropterus* Zunino & Halffter, 1981), all of them endemic, show wing reduction (Howden & Young, 1981; Solís et al., 2024). Costa Rica mountain ranges (especially Talamanca) are also the areas with the highest brachypterous species (7) and generic density (4) in America (Kohlmann, 2022; Kohlmann et al., 2023). A similar mechanism involving high levels of endemicity and brachypterism has been reported for the Passalidae (Coleoptera: Scarabaeoidea) by Jiménez‐Ferbans et al. (2017) for the Costa Rican mountains, especially the Talamanca Range. All these facts suggest a scarab Miocene colonization and endemic island speciation processes in Costa Rica.

### 2.4 Distribution

Four previously proposed dung beetle biogeographical distribution patterns were studied for the present analysis (Solís & Kohlmann, 2004):

1. Endemic Costa Rican species (NCP), few shared with Nicaragua and some with Panama. Phylogenetic analyses like the one undertaken by Breeschoten et al. (2016) on *Onthophagus* and Gillett and Toussaint (2020) on phanaeine dung beetles point to a Miocene origin of these endemic species (15–5 Mya). Contrastingly, Moctezuma et al. (2024): figure 2) propose an early origin of 5–1.5 Mya for the endemic phanaeine species. The estimation for the origin of endemic phanaeine species by Moctezuma et al. (2024) conflicts with the geological information of the area (Solís et al., 2024). The time of origin proposed by Moctezuma et al. (2024) would mean that Costa Rica was already part of North America and did not partake of the intense endemicity generating process characteristic of an island syndrome. Lizardo et al. (2024) cite Costa Rica as the area in all America with the highest species richness of taxa with conserved temperature niches and no species with shifted adaptive temperature regimes. Lower Central American (Costa Rica and Panama) lineages did also expand to North and South America, as has been suggested for the bess beetles (Passalidae) and *Onthophagus* of the clypeatus‐dicranius species groups (Beza‐Beza et al., 2021; Kohlmann, 2022).
2. Species whose distribution is located between Mexico and Panama (MP). This pattern contains different waves of expansion processes like the invasion of tropical dry forest originating in Mexico around 2.5 Ma (Becerra, 2005; Graham & Dilcher, 1995; Pérez‐García et al., 2012) and recent colonization mediated by the Last Glacial Maximum (LGM) around 20 ka last (Jackson et al., 2019; MacVean & Schuster, 1981).
3. Species whose distribution goes from South America reaching Costa Rica and rarely Nicaragua (NSA). This limited distribution pattern in Central America points to a recent northward expansion of South American elements into Central America during the last 10,000 years (Burnham & Graham, 1999; Colinvaux, 1997; DeVries, 1987). Moctezuma et al. (2024): figure 2 suggest a possible time origin of the species *Canthidium guanacaste* around 14 Mya. However, this estimation is not coeval with the appearance of the tropical dry forest in Costa Rica (see above).
4. Extensively distributed species stretching from Mexico to South America (NASA). This distribution is suggestive of a two‐way Miocene or pre‐Miocene island‐hopping expansion between North and South America, later augmented and facilitated by the closure of the Isthmus of Panama around 2.8 Mya (Coates, 1997; Montes et al., 2015; O'Dea et al., 2016; Solís et al., 2024). Magalhaes et al. (2023) cite an intensive, bidirectional, and asymmetric (chiefly South to North) *Micrathena* spider's species exchange between Central and South America at least 20 Mya, where most of the dispersal events (63%) predate formation of the Isthmus of Panama.

At the same time, three previously suggested (Kohlmann & Wilkinson, 2007; Kohlmann) geographical basins/regions were studied: the Caribbean, the North Pacific, and the South Pacific. Fogden and Fogden (1997) had previously proposed a similar broad‐scale vegetation biogeographical scheme.

### 2.5 Data analyses

To assess the effect of altitude on *Onthophagus* and *Canthidium* species richness, we performed polynomial models, which was more appropriate due to the non‐linear trend observed in our data and other altitudinal gradient studies in the Neotropical region (e.g., Alvarado et al., 2020; da Silva et al., 2018). Altitude was the predictor variable, and species richness was the response variable. For the analysis, we tested the effect of altitude on each genus separately. Data was analyzed in R software version 4.1.3 (R Development Core Team, 2022).

We assessed the effect of species biogeographical distribution patterns (Mexico to Panama, MP; Nicaragua to Panama, NCP; North America to South America, NASA; Nicaragua to South America, NSA) and regions (Caribbean, North Pacific, South Pacific) on altitudinal mean distribution and altitudinal range. Species distribution patterns and regions were the predictor variables, while species' altitudinal mean distribution and altitudinal range were the response variables. Analyses were performed for *Canthidium* and *Onthophagus*, and each genus was analyzed separately. Due to the limited number of samples (i.e., species), which could bias the statistical results, we removed the data of NSA in the species distribution patterns model performed exclusively with *Onthophagus* (*s* = 3). We also removed NASA and NSA data for the model performed exclusively with *Canthidium* (*s* = 4 and 3, respectively). Likewise, for the models that assessed the effect of region, we removed North Pacific data from the model performed exclusively with *Canthidium* (*s* = 3 for this region). Data were analyzed by using Linear Models and Generalized Linear Models with Negative Binomial (when data showed overdispersion – Residual deviance/Residual d. f. > 2) in R software (R Development Core Team, 2022). Generalized Linear Models with Negative Binomial analyses were performed in the MASS package (Ripley et al., 2023).

Whenever we had a low number of outliers (*n* < 2), they were removed from the analyses. For models encompassing many outliers, we made model comparisons using Residual Standard Error, aiming to find the one that best‐fitted data distribution. Thus, *in Onthophagus's* analyses (both for the effects of species distribution pattern and region), the response variable ‘altitudinal range’ was analyzed by using Robust Linear Regression to control the bias caused by the outlier data or with linear models when such model had a better (i.e., lower) Residual Standard Error. To obtain statistical *p* values of robust linear regressions, we performed a robust *F*‐test. Robust linear regression and robust *F*‐tests were conducted in MASS and SFSMISC packages (Maechler et al., 2023; Ripley et al., 2023).

## 3 RESULTS

### 3.1 Species diversity

A total of 71 species were studied, 41 *Onthophagus* and 30 *Canthidium* (Table 1, Appendix S1). The South Pacific was the Costa Rican region with the highest number of species (26 *Onthophagus*, 18 *Canthidium*), closely followed by the Caribbean region (21 *Onthophagus*, 21 *Canthidium*), with the North Pacific being the less speciose one (15 *Onthophagus*, 3 *Canthidium*) (Table 1). Regarding dung beetle biogeographical distribution, most Costa Rican species were endemic to the country (NCP, *Onthophagus s* = 23, *Canthidium s* = 19), followed by species with a distribution ranging from Mexico to Panama (MP, *Onthophagus s* = 9, *Canthidium s* = 5). Species that ranged from North to South America (NASA) and from Costa Rica/Nicaragua to South America (NSA) were the less speciose groups (*Onthophagus s* = 6 and 3, *Canthidium s* = 3 and 3, respectively) (Figure 3). Endemic species were dominant in all geographic regions at all altitudinal bands except for the 0–1000 m a.s.l. band for the North and South Pacific regions, where the MP and NASA biogeographic distribution patterns were dominant and codominant, respectively (Table 1). Dung beetle species had a mean body size of 6.04 mm, whereas *Canthidium* species (mean of 4.87 mm) were smaller than *Onthophagus* (mean of 6.79 mm, see Appendix S1 in Supplementary Information).

**TABLE 1:** Number of species and endemics of *Canthidium* and *Onthophagus* by altitudinal band in continental Costa Rica (Modified from Kohlmann & Wilkinson, 2007).

Region	Altitudinal band m a.s.l.	Area km^2^	Species number		Endemic species number		Species per distribution pattern			
			C	O	C	O	1	2	3	4
Caribbean	0–1000	18,513	14	20	6	10	16	8	3	7
	1000–2000	4005	17	14	11	6	17	7	1	6
	>2000	1740	4	1	4	0	4	1	0	0
	Subtotal	24,258	21	21	13	11	24	8	3	7
South Pacific	0*–*1000	9767	12	16	4	4	8	6	5	9
	1000*–*2000	2451	16	21	9	10	19	8	2	8
	>2000	930	3	10	3	7	10	3	0	0
	Subtotal	13,145	18	26	10	12	22	8	5	9
North Pacific	0*–*1000	12,034	3	10	0	2	2	6	1	4
	1000*–*2000	1495	0	12	0	5	5	4	0	3
	>2000	103	0	7	0	4	4	3	0	0
	Subtotal	13,632	3	15	0	5	5	8	1	4
	Total	51,038	30	41	19	23	42	14	6	9

*Note*: Number of species per biogeographic distribution pattern: 1 = NCP, 2 = MP, 3 = NSA, 4 = NASA.

Abbreviations: C, *Canthidium*; O, *Onthophagus*.

### 3.2 Altitudinal diversity gradient

**FIGURE 2:**
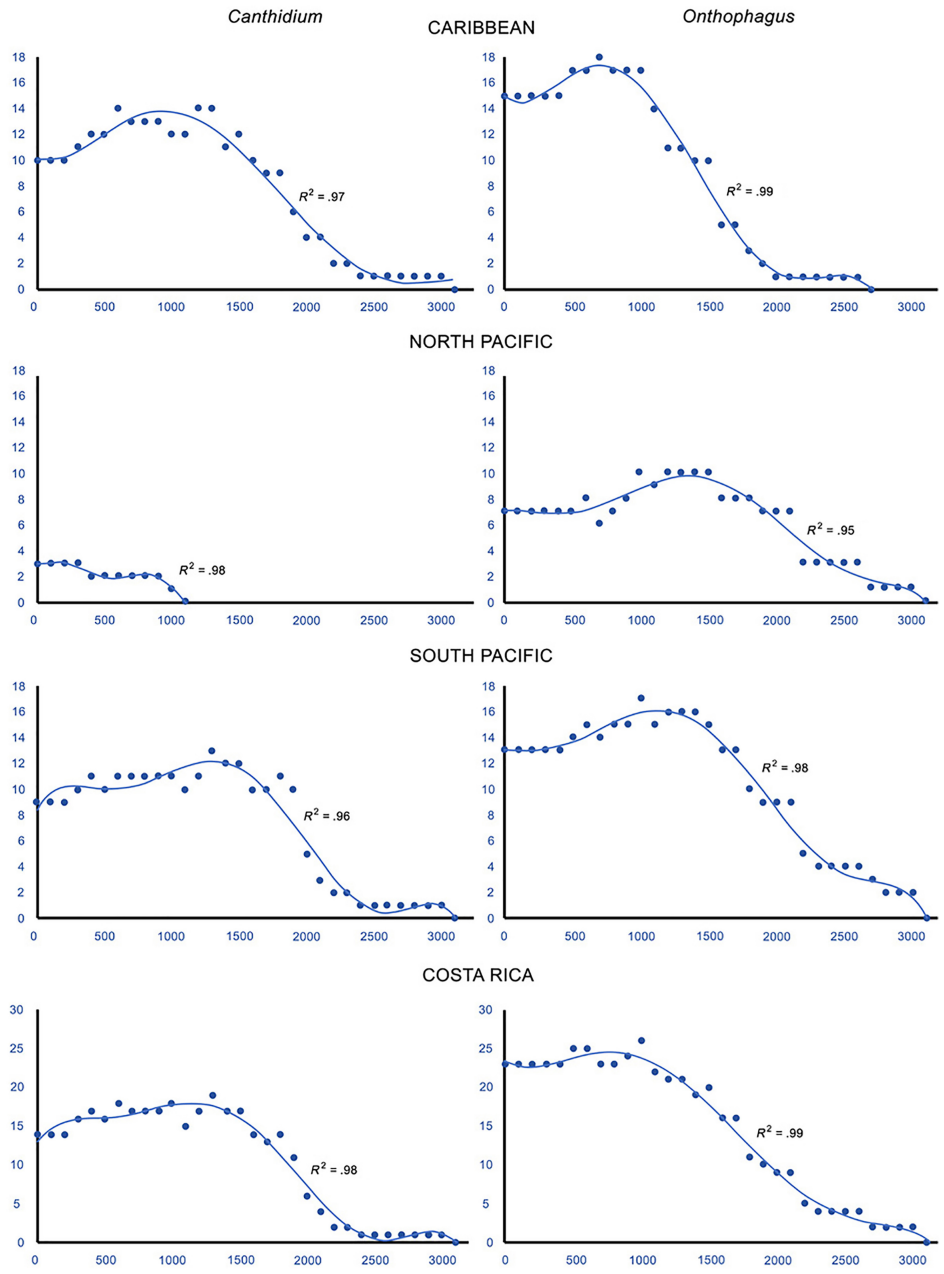
*Canthidium* and *Onthophagus* species biodiversity curves along an altitudinal gradient for different regions of Costa Rica.

Costa Rican *Onthophagus* and *Canthidium* species are altitudinally distributed from 0 m a.s.l. to ca. 3000 m a.s.l. (Figure 2). The peak of species richness varied between genera, but there was a general trend for an extremely reduced number of species at high altitudes. For both *Onthophagus* and *Canthidium*, species richness tended to decrease with increasing altitude (Figure 2). When considering *Onthophagus* species (*F*_4,26_ = 356.43; *p* < .01), species richness presented an abrupt decline after 1000 m a.s.l. (Figure 2), while this strong decline was observed for *Canthidium* species (*F*_5,25_ = 125.54; *p* < .01) at altitudes higher than 1500 m a.s.l. (Figure 2).

**FIGURE 3:**
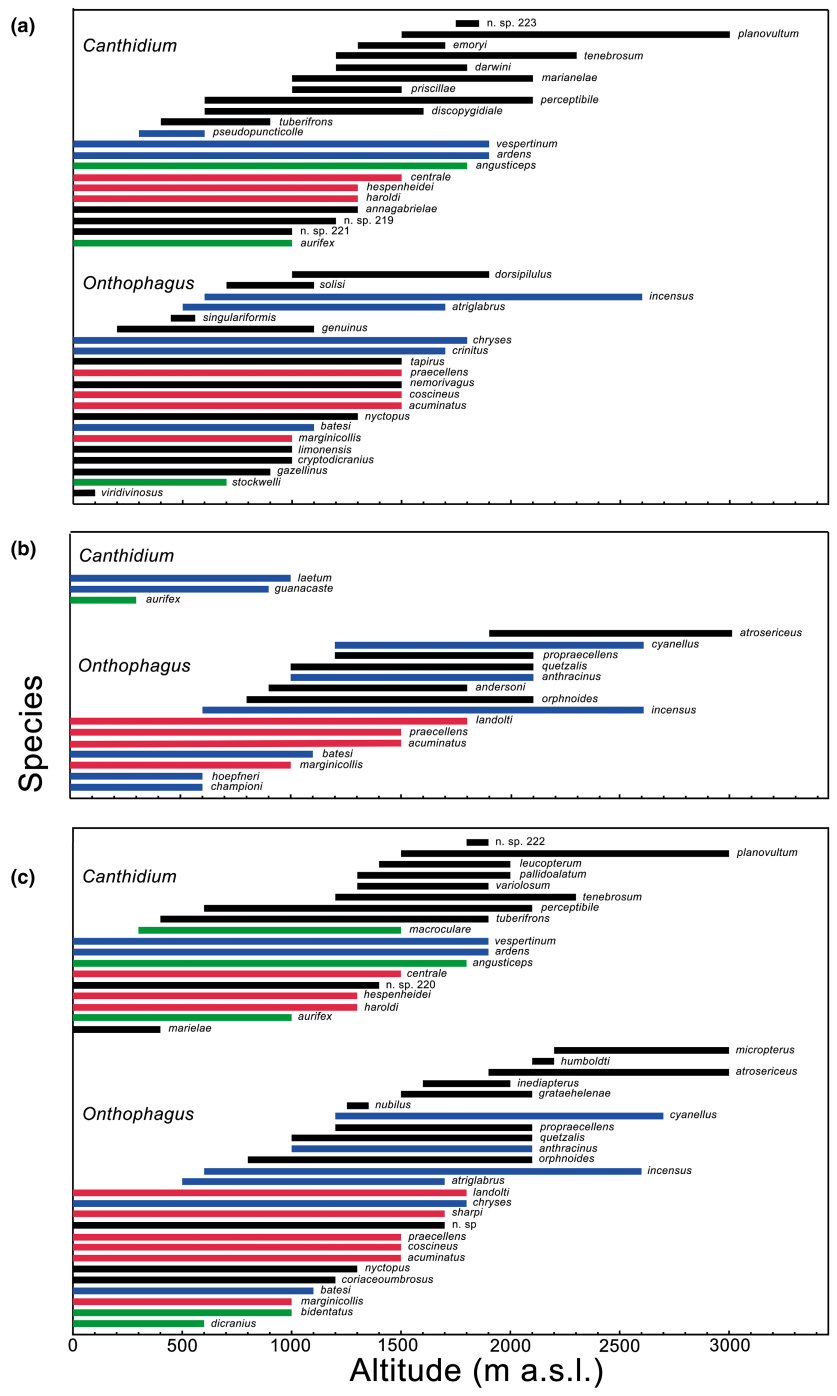
Figure showing the distribution of *Canthidium* and *Onthophagus* species throughout different geographic regionsw (a Caribbean, b North Pacific, c South Pacific), along an altitudinal gradient in Costa Rica. The following biogeographic distribution patterns are indicated: MP = from Mexico to Panama (blue); NASA = from Mexico to South America (red); NCP = Costa Rica endemics (Nicaragua to Panama) (black); NSA = from Costa Rica/Nicaragua to South America (green). All species that start their distribution at 0 m a.s.l. are lowland species, those that do not are mountain ones.

For both genera, maximum diversity was observed below the middle of the gradient. Then, the curve slopes down, reaching a minimum of around 3000 m a.s.l. (Figure 2). *Onthophagus* in the North Pacific presents an altitudinal diversity peak (10) from 1200 to 1500 m a.s.l. In contrast, in the South Pacific, it registers an altitudinal diversity peak (16) from 1200 to 1300 m a.s.l. and the Caribbean (17) from 600 to 900 m a.s.l. (Figure 3). For *Canthidium*, the North Pacific registers an altitudinal diversity plateau (3) from 0 to 300 m a.s.l. In contrast, the Caribbean has an altitudinal diversity peak (13) for *Canthidium* from 600 to 900 and from 1200 to 1300 m a.s.l. and the South Pacific (11) from 1200 to 1500 m a.s.l. It is clear from Table 1 that both genera do not follow an island species‐area relationship (ISAR).

### 3.3 Biogeographic distribution patterns and geographic regions

**FIGURE 4:**
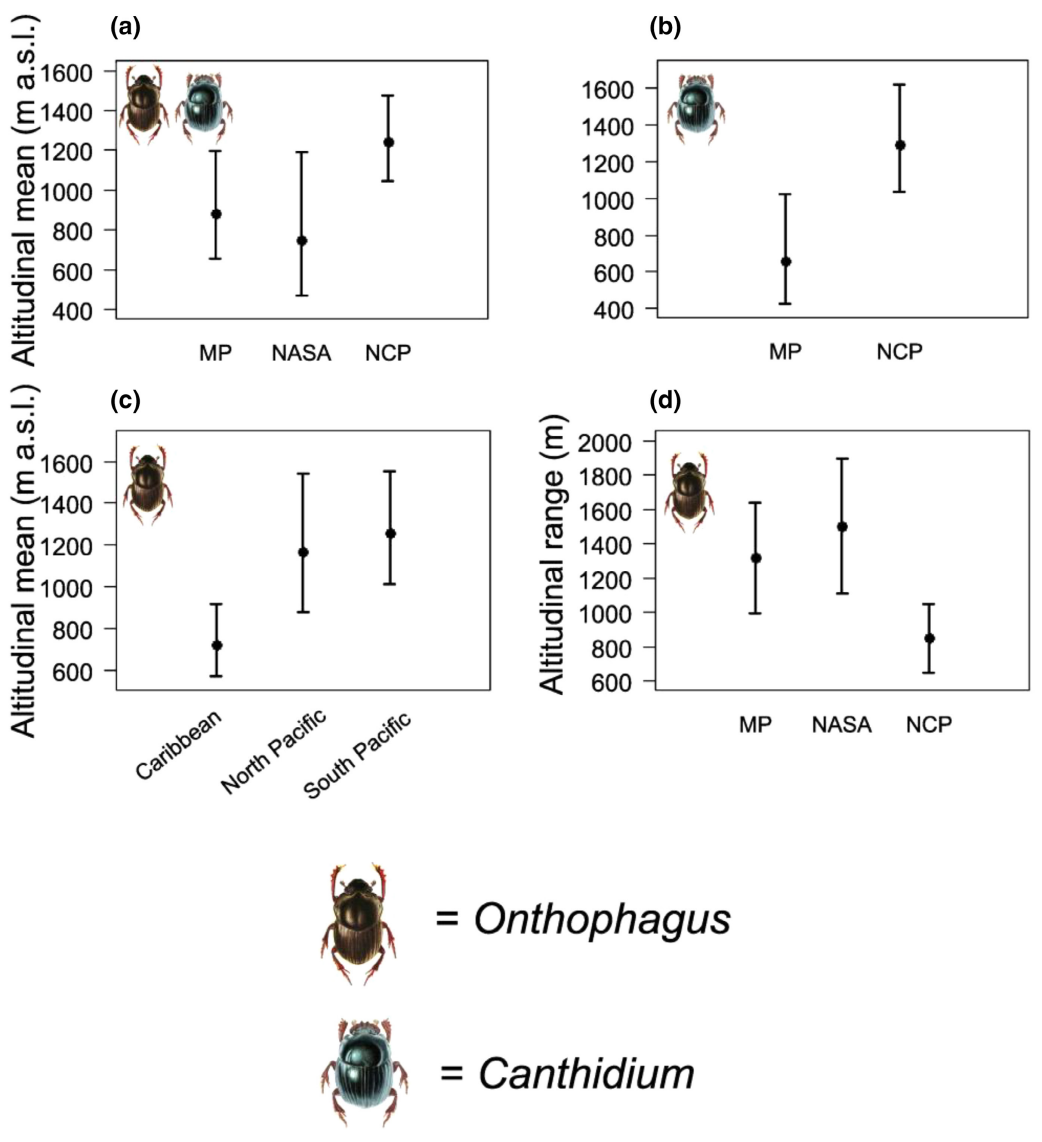
Statistically significant effects of *Canthidium* and *Onthophagus* biogeographic distribution patterns and geographic regions on dung beetle altitudinal means (a–c), and effects of distribution patterns on altitudinal range (d). MP = from Mexico to Panama; NASA = from Mexico to South America; NCP = Costa Rica endemics (Nicaragua to Panama); NSA = from Costa Rica/Nicaragua to South America.

Dung beetle biogeographic distribution patterns and geographic regions affected the species' altitudinal means and ranges (Table 2). Most endemic species (NCP) are mountain species (Figure 3), thus resulting in a higher altitudinal mean when compared to the other distribution patterns (Figure 4a). For *Canthidium*, species distribution patterns affected its altitudinal mean, where the endemic species (NCP) register a higher altitudinal mean (Figure 4b, Table 2). The geographic regions showed differences in the spatial distribution of *Onthophagus* beetles, where the Caribbean region records a lower altitudinal mean than the North and South Pacific regions (Figure 4c, Table 2). The *Onthophagus* endemic species (NCP) presented a significantly reduced altitudinal range variation concerning other *Onthophagus* biogeographic distributions (Figure 4d, Table 2).

**TABLE 2:** Statistical effects of distribution pattern, region and body size on species altitudinal range, altitudinal mean, and body size of *Onthophagus* and *Canthidium* dung beetles from Costa Rica.

	Distribution pattern		Region		Body size
	Distribution pattern	Distribution pattern: Body size	Region	Region: Body size	
Both genera					
Body size	*F*(2, 56) = 0.07; *p* = .92	NA	***F*(3, 97) = 2.88; *p* = .03**	NA	NA
Altitudinal range	*F*(2, 59) = 2.50; *p* = .09	*F*(3, 55) = 1.81; *p* = .15	*F*(3, 100) = 0.41; *p* = .73	*F*(4, 96) = 0.54; *p* = .70	*F*(1, 57) = 0.27; *p* = .59
Altitudinal mean	***χ*** ^**2**^ **(2, 59) = 65.30; *p* = .04**	*χ* ^2^(3, 55) = 62.18; *p* = .13	*χ* ^2^(3, 100) = 109.33; *p* = .08	***χ*** ^**2**^ **(4, 96) = 106.04; *p* = .03**	*χ* ^2^(1, 57) = 62.42; *p* = .73
*Onthophagus*					
Body size	*F*(2, 35) = 2.21; *p* = .12	NA	*F*(2, 59) = 0.31; *p* = .73	NA	NA
Altitudinal range	**NCP:** ***F* = 5.73; *p* = .02** NASA: *F* = 0.52; *p* = .47	***F*(3, 34) = 5.83; *p* < .01**	North Pacific: *F* = 0.01; *p* = .90 South Pacific: *F* = 0.01; *p* = .91	Caribbean: *F* = 0.33; *p* = .56 North Pacific: *F* = 0.17; *p* = .68 South Pacific: *F* = 0.27; *p* = .60	*F* = 0.07; *p* = .78
Altitudinal mean	*χ* ^2^(2, 35) = 40.29; *p* = .25	*χ* ^2^(3, 34) = 40.28; *p* = .38	***χ*** ^**2**^ **(2, 59) = 65.08; *p* < .01**	***χ*** ^**2**^ **(3, 58) = 64.82; *p* < .01**	*χ* ^2^(1, 36) = 40.37; *p* = .27
*Canthidium*					
Body size	*F*(1, 19) = 0.46; *p* = .50	NA	*F*(1, 34) = 0.02; *p* = .85	NA	NA
Altitudinal range	*F*(1, 22) = 0.59; *p* = .44	*χ* ^2^(2, 18) = 25.00; *p* = .62	*χ* ^2^(1, 37) = 45.02; *p* = .69	*χ* ^2^(3, 33) = 41.72; *p* = .68	*χ* ^2^(1, 19) = 25.00; *p* = .54
Altitudinal mean	***χ*** ^**2**^ **(1, 22) = 24.89; *p* < .01**	*χ* ^2^(1, 17) = 21.56; *p* < .10	*χ* ^2^(1, 37) = 40.52; *p* = .65	***χ*** ^**2**^ **(2, 33) = 37.20; *p* = .05**	***χ*** ^**2**^ **(1, 18) = 20.71; *p* < .01; *R*** ^**2**^ **= .33 (−)**

*Note*: Statistically significant effects are shown in bold.

Abbreviation: NA, statistical model not applicable.

### 3.4 Body size

**FIGURE 5:**
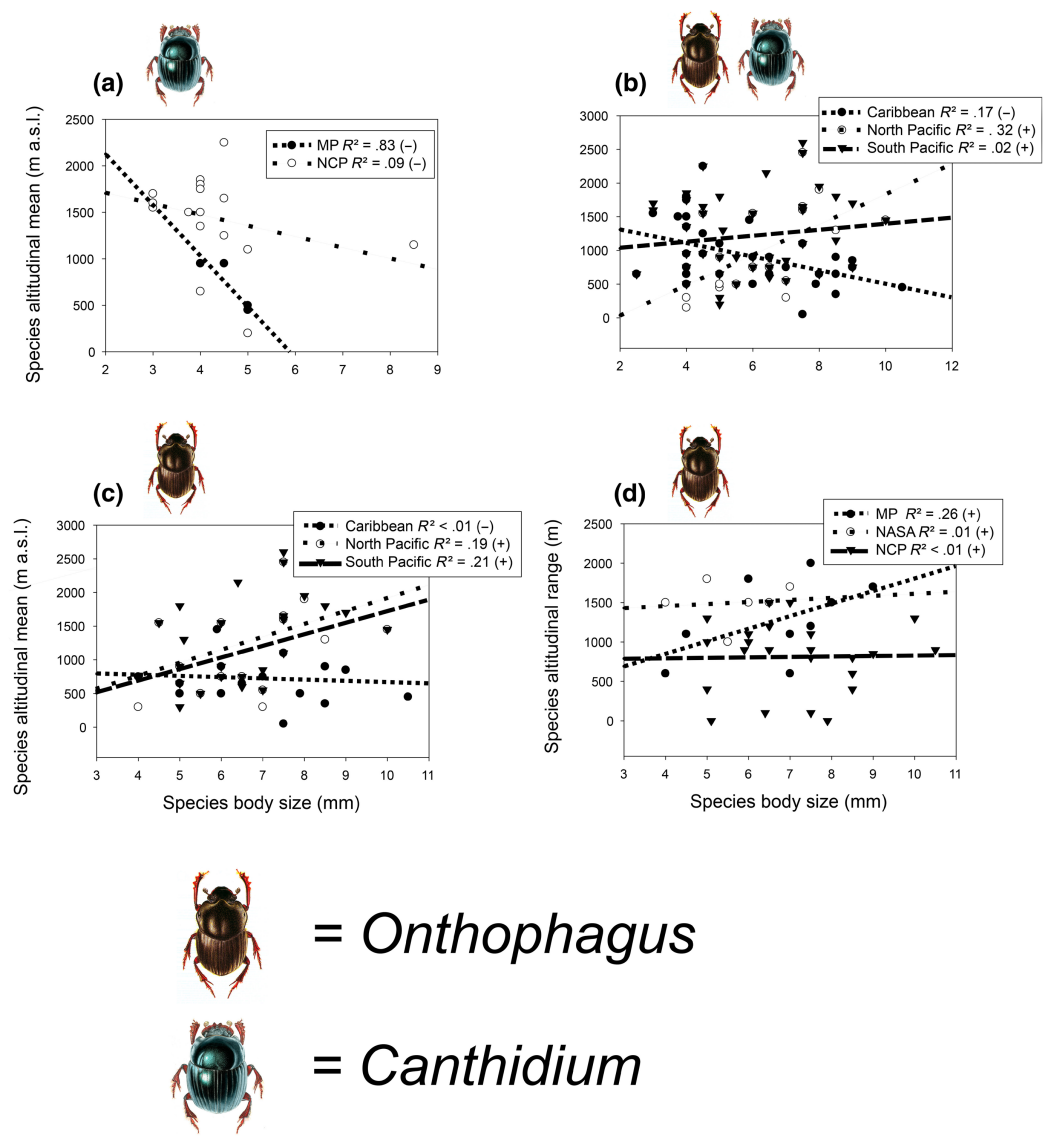
Statistically significant effects of the interaction of body size and biogeographic distribution patterns and body size and geographic regions affecting altitudinal means (a–c) and effects of body size and distribution patterns on altitudinal ranges (d) of *Canthidium* and *Onthophagus* species of Costa Rica. MP = from Mexico to Panama; NASA = from Mexico to South America; NCP = Costa Rica endemics (Nicaragua to Panama); NSA = from Costa Rica/Nicaragua to South America.

*Canthidium* presented a statistically negative relationship between body size and altitudinal mean (Table 2). Also, there was a statistically significant interaction between body size and distribution pattern affecting the altitudinal mean (Table 2, Figure 5a), where the Mexico to Panama (MP) and the endemic (NCP) species show a strong and weak negative relationship, respectively (Figure 5a). When analyzing both genera together, body size and geographic region affect the altitudinal mean (Table 2, Figure 5b), where the North and South Pacific register a positive trend and the Caribbean a negative one. For *the Onthophagus* there were statistically significant interactions between geographic regions and body size affecting the altitudinal mean (Table 2, Figure 5c). The North and South Pacific show a positive trend, while the Caribbean a weak negative one (Figure 5c). *Onthophagus* also registers statistically significant interactions between distribution patterns and body size affecting altitudinal ranges (Table 2, Figure 5d). Endemic species (NCP), as well as North America to South America (NASA) species show a weak positive pattern, whereas a strong positive one is shown by the Mexico to Panama (MP) taxa (Figure 5d).

## 4 DISCUSSION

### 4.1 Species diversity and geology

Costa Rica records the highest species density in the American Continent for *Canthidium* and *Onthophagus*, with 0.6 species/1000 km^2^ and 0.8 species/1000 km^2^, respectively; as well as having the highest dung beetle species and endemics density in continental America (3.6 and 0.8 per thousand km^2^, respectively; Cupello et al., 2023). In second place, we have Panama with a *Canthidium* species density of 0.3 species/1000 km^2^ and Oaxaca state (located in Mexico) with an *Onthophagus* species density of 0.5 species/1000 km^2^ (Kohlmann et al., 2023). These three areas have been well‐sampled, but other Central American countries like El Salvador and Honduras have been poorly examined, and their relatively low species diversity could be related to sampling artifacts. However, what is truly remarkable is the enormous presence of endemic dung beetle species (NCP) for both genera, 63% for *Canthidium* (19 spp.) and 56% for *Onthophagus* (23 spp) (Table 1). For instance, Oaxaca, the most biodiverse Mexican state and arguably comparable to Costa Rica in extension and orography, records 41% and 34% of endemic *Canthidium* (5 spp.) and *Onthophagus* (17 spp.) species, respectively (Kohlmann et al., 2023; Moctezuma et al., 2023). Ecuador registers 28% for endemic *Canthidium* (5 spp.) and 13% for endemic *Onthophagus* (3 spp.) (Chamorro et al., 2019). Costa Rican endemicity numbers advocate an insular evolutionary process during the genesis of the region, resulting in a high number of autochthonous species for both genera.

*Onthophagus* and *Canthidium* endemic percentages in Costa Rica are comparable with insular systems, although one can expect that in the case of Costa Rica the percentages have been diluted due to subsequent invasions of North and South American taxa. In the Greater Antilles, for example, 45 species of dung beetles have been recorded, all of them endemics (Génier & Howden, 2014; Ivie & Philips, 2008; Matthews, 1966; Philips & Ivie, 2008). The following dung beetle endemic percentages have been calculated by Cupello et al. (2023): Cuba 77%, Dominican Republic 73%, Jamaica 67%, and Puerto Rico 86%. Costa Rica compares well to the Greater Antilles regarding dung beetle endemics percentages.

It is a well‐known fact that a typical island syndrome is the presence of high numbers of endemic species (Lomolino et al., 2017; Matthews & Triantis, 2021; Whittaker et al., 2017, 2023). Islands represent less than 10% of the Earth's surface but contain almost 50% of all endemic bird species (Beierkuhnlein, 2007). Isolated islands report high percentages of endemics, as are the cases for plants in the Azores (34%), Cuba (50%), Galápagos (45%), Madagascar (87%), New Caledonia (75%), New Zealand (85%), Japan (27%), and Saint Helena (69%) (Whittaker et al., 2023).

### 4.2 Altitudinal diversity gradient

For both genera, maximum diversity was observed below the middle of the gradient (Figure 2). Most altitudinal diversity curves are unimodal and positively skewed (Guo et al., 2013). In addition, most of the diversity peaks are located lower than the mid‐elevation of the sampled gradient (Guo et al., 2013). The present analysis followed all these general patterns. However, not all altitudinal diversity curves in Costa Rica follow this arrangement in the case of other taxonomic groups. Fallas (2015) cites a unimodal negative skew for moths, a mid‐domain effect for epiphytes, and a negative gradient for ants. These differences can be a function of the diversity of the taxonomic groups, climatic variables, sampling methodologies, mountain range configuration, area, physiological differences, and anthropogenic disturbances (Kohlmann et al., 2021). Escobar et al. (2005) and Lizardo et al. (2022) undertook an analysis of Scarabaeinae assemblages of the Eastern Colombian Andes and a continental analysis of the distribution of the Phanaeini, respectively. As is here also the case, maximum diversity was observed below the middle of the gradient, the species curve is unimodal and positively skewed, and biodiversity diminished rapidly above 2000 m a.s.l. Villamarín‐Cortez et al. (2022) report for Ecuador a reduction of biodiversity for Canthonini, Coprini, and Phanaeini with an increase of altitude. Although our study represents a particular gradient (a watershed altitudinal gradient) when compared to the abovementioned ones (continental gradients and mountain chain altitudinal gradients), our data and those presented by previous research coincide in that they report dung‐beetles to be infrequent over 2000 m a.s.l.

*Canthidium* presents a particular diversity case in the North Pacific. Its altitudinal diversity curve did not follow a unimodal pattern but a two‐stepped staircase model (Figures 2 and 3). The tropical dry Pacific Forest represents a relatively new environment that *Canthidium* colonized when it spread to North America. As indicated before, the tropical dry forest spread from Mexico into Costa Rica around 2.5 Mya (Becerra, 2005; Graham & Dilcher, 1995; Pérez‐García et al., 2012). Currently, it registers in Costa Rica two native dry tropical forest species (*C. guanacaste* Howden and Gill, 1987; *C. laetum* Harold, 1867) and a fringe penetration by one species (*C. aurifex* Bates, 1887) at the Caribbean border area (Figure 3). No *Canthidium* species are present in the North Pacific mountains. In total, only three native species have been recorded for the whole extension of the tropical dry Pacific Forest stretching from Mexico to Costa Rica (Solís & Kohlmann, 2004), suggesting that speciation by *Canthidium* in this environment has been a recent or a slow affair.

There is a marked tendency in Costa Rica for endemic species (NCP) to be mountain dwellers (1000–1450 m a.s.l.), presenting a small altitudinal range variation of approx. 400 m (Tables 1 and 2, Figure 4a). Nowhere is this more evident than in the case of the North Pacific, where all endemic *Onthophagus* species are upland taxa (Figure 3). This distribution suggests that endemic species have followed a Costa Rican upland speciation strategy, probably trying to avoid aggressive lowland invaders and/or possibly trying to fill new unoccupied niches as the Talamanca Mountain range arose during the Late Miocene (8–5 Mya).

However, these endemics never developed specialized high mountain lineages, such as the *Onthophagus chevrolati* species complex did in the Mexican‐Northern Central American mountains, registering around 50 montane taxa (Halffter et al., 2019; Zunino & Halffter, 1988), thus evincing an upper mountain faunal deficiency. Since the *Canthidium* and *Onthophagus* Costa Rican colonizers were lines adapted to an original tropical environment, especially in the case of the *O. clypeatus* and *O. dicranius* species groups which are assumed to be of boreotropical origin (Kohlmann, 2022), they probably needed more time for such an evolutionary feat. Still, they managed to generate some endemic montane species (*O. atrosericeus* Boucomont, 1932; *O. humboldti* Kohlmann, Solís and Alvarado, 2019; *O. micropterus* Zunino & Halffter, 1981; *C. planovultum* Howden & Young, 1981; Figure 3).

We can compare the four upper montane (≥2500 m a.s.l.) *Onthophagus* species, a mountain‐adapted genus, from Costa Rica (*O. atrosericeus*, O. cyanellus*, O. incensus*, *O. micropterus*; * MP, species that most probably dispersed from Mexico to Panama; Figure 3) to the 10 species reported from Oaxaca, Mexico (≥2500 m a.s.l.; Escobar‐Hernández et al., 2019; Salomão et al., 2021). Only two Costa Rican upper montane taxa versus 10 Oaxacan montane taxa, and two other upper montane Costa Rican species that most probably dispersed from Mexico during a glaciation process.

Middle American species (MP) are evenly distributed in the up‐and‐lowlands (Figure 3). The lowlands are occupied mainly by taxa with broad geographic distributions in North and South America or having spread from South America in a limited way into Central America (NASA, NSA; Figure 3). The lowlands are largely colonized by species that originated elsewhere, probably helped in their expansion when insular Costa Rica connected with the mainland during the Pliocene. These two distribution patterns, lowland with broad geographic distributions and montane endemic with limited distributions, have also been reported by Beza‐Beza et al. (2023) for bess‐beetles (Passalidae) in Central America. These authors report that the transition between these two distributions occurs at approximately 1500 m a.s.l. In the present study, both distribution patterns overlap from 500 to 1500 m a.s.l.

### 4.3 Biogeographic distribution patterns and geographic regions

Biogeographic distribution patterns and geographical regions determined dung beetle species' altitudinal means and ranges (Table 2, Figure 4). *Canthidium* and *Onthophagus* endemic species (NCP) are chiefly composed of upland taxa, whereas extensively distributed species (MP, NASA) are essentially lowland taxa and possibly represent invaders from other areas. As indicated above, these endemic species could have tried to avoid competition with aggressively invading lowland species by colonizing and speciating emerging mountain ranges.

Interestingly, the endemic *Onthophagus* species registered a small altitudinal range. The slight range variation can be explained by the following process reported by Kohlmann et al. (2021) for *Onthophagus* living in North American tropical mountains. Kohlmann et al. (2021) suggest that the severity of a mountain glaciation can influence the Elevational Rapoport Rule (ERR; Stevens, 1992), resulting in an incomplete postglacial mountain recolonization, given the limited time for dispersal since the LGM. This mechanism could be operating for the *Onthophagus* species living on the Talamanca Range in the Caribbean and South Pacific. This mountain range suffered its last glacial maximum ~20 ka ago (Jackson et al., 2019). During that time, the treeless *páramo* extended down to 2100 m altitude during the last glacier interval, whereas it is distributed from 3300 to 3819 m a.s.l. at present (Horn, 2007). It is, therefore, possible that these endemic mountain species are still in the process of expanding upwards.

### 4.4 Body size

Dung beetle species' body size, geographic regions, and biogeographic patterns interacted significantly with altitudinal means and ranges (Table 2, Figure 5). Body size follows two distinct evolutionary avenues in Costa Rica. Endemic species (NCP) follow Foster's Rule, also known as the Island Rule or Island Effect (Insular Dwarfism, Insular Gigantism), where the endemic species of *Canthidium* are smaller than the non‐endemic ones, and the endemic species of *Onthophagus* are larger than the non‐endemic ones (Solís et al., 2024). The endemic *Onthophagus* species becoming larger than the non‐endemic ones is the first recorded instance of such behavior in an island dung beetle group.

Gillett and Toussaint (2020) have reported that Phanaeini (*Coprophanaeus* Olsoufieff, 1924 and *Phanaeus Macleay*, *1819*) endemic dung beetle taxa that speciated in Costa Rica during the middle to late Miocene (~14 to ~9 Ma) also show body size reduction in comparison to non‐endemic species. Along the same line and in a very interesting study done by de Cerqueira et al. (2023) of islands created by the Balbina Hydroelectric Reservoir constructed in 1987 in the Central Amazon, it is reported that body‐size dung beetle responses to insularization were species‐dependent. They indicated for example, that larger islands tended to host larger individuals of *Deltochillum aspericole* Bates, 1870, while smaller islands showed larger body sizes in *Canthon triangularis* (Drury, 1770). Still, individuals from the mainland were larger than those from the islands (de Cerqueira et al., 2023).

The body‐size behavior of the Middle American (MP) *Onthophagus* species (Figure 5d) can be explained by the Elevational Rule of Rapoport, where large‐body upland‐dwelling species present wider altitudinal ranges than small‐body taxa from lower altitudes. This body‐size behavior supports the idea that these upland (MP) species are represented in Costa Rica by taxa that speciated in the Mexican‐Guatemalan mountains. This biogeographical context suggests the maintenance of a wide altitudinal range and a large body size, thus conserving their original niche (Salomão et al., 2021). Neither endemic (NCP) nor extensively distributed species (NASA) seem to follow this pattern.

## 5 CONCLUSIONS

The *Canthidium* and *Onthophagus* fauna in Costa Rica are represented by different biogeographic and evolutionary lineages intertwined with different colonization times and waves, suggesting that this area has a composite historical character. Based on attributes mentioned earlier, Costa Rica fits better the description of De Mendonça and Ebach (2020) that the country represents a geographic overlap of two or more temporally disjunct areas and is not part of a natural transition zone.

*Canthidium* and *Onthophagus* show differences and similarities in their altitudinal distribution patterns, suffused by island syndrome processes. *Canthidium* and *Onthophagus* altitudinal diversity curves present different shapes depending on the biogeographic origin of the ecosystem in question. Both genera have brachypterous species and follow Foster's Rule, *Canthidium* towards dwarfism and *Onthophagus* towards gigantism. *Both genera* present lowland species with broad geographic distributions and numerous mountain microendemics, most probably generated by their insular origin.

Costa Rica's baffling biodiversity has been usually explained by other authors (Burger, 1980; DeVries, 1987; Fogden & Fogden, 1997; Savage, 2002) as an interplay of high mountain ranges between two oceans, two continents, and two lowlands; the richness of volcanic soils; and seasonal precipitation patterns. Glaciations have also been considered a dynamic biodiversity‐generating system (Hooghiemstra et al., 1992). Only Stehli and Webb (1985) have considered the geologic origin of Costa Rica as an archipelago as a possible biodiversity‐driving engine. One could argue that the effect of the insular Miocene origin of Costa Rica still pervades today, contributing to its enormous present‐day biodiversity and the different insular syndrome behaviors of its dung beetle fauna (brachyptery, very high endemicity levels, microendemisms, compliance to Foster's Rule, upper montane faunal deficiency, dietary shifts).

## AUTHOR CONTRIBUTIONS

**Bert Kohlmann:** Conceptualization (lead); data curation (equal); investigation (lead); methodology (equal); project administration (equal); supervision (equal); visualization (equal); writing – original draft (lead); writing – review and editing (equal). **Renato Portela Salomão:** Data curation (equal); formal analysis (lead); methodology (equal). **Ángel Solís:** Conceptualization (equal); data curation (equal); investigation (equal); project administration (equal).

## FUNDING INFORMATION

No funding was received.

## CONFLICT OF INTEREST STATEMENT

The authors have no conflict of interest to report.

## Supporting information


Appendix S1.

## Data Availability

All data are presented as supplementary files.
